# The Burden of Health-Related Out-of-Pocket Cancer Costs in Canada: A Case-Control Study Using Linked Data

**DOI:** 10.3390/curroncol29070359

**Published:** 2022-06-27

**Authors:** Beverley M. Essue, Claire de Oliveira, Tracey Bushnik, Sharon Fung, Jeremiah Hwee, Zhuolu Sun, Elba Gomez Navas, Jean Hai Ein Yong, Rochelle Garner

**Affiliations:** 1 Institute of Health Policy, Management and Evaluation, Dalla Lana School of Public Health, University of Toronto, Toronto, ON M5T 3M7, Canada; claire.deoliveira@utoronto.ca; 2 Canadian Partnership Against Cancer, Toronto, ON M5H 1J8, Canada; sharon.fung@partnershipagainstcancer.ca (S.F.); zhuolu.sun@partnershipagainstcancer.ca (Z.S.); elba.gomez@partnershipagainstcancer.ca (E.G.N.); jean.yong@partnershipagainstcancer.ca (J.H.E.Y.); 3 Centre for Health Economics and Hull York Medical School, University of York, Heslington, York YO10 5DD, UK; 4 Centre for Addiction and Mental Health, Institute for Mental Health Policy Research, Toronto, ON M6J 1H4, Canada; 5 Health Analysis Division, Statistics Canada, Ottawa, ON K1A 0T6, Canada; tracey.bushnik@canada.ca (T.B.); jeremiah.hwee@mail.utoronto.ca (J.H.); rochelle.garner@statcan.gc.ca (R.G.)

**Keywords:** health-related out-of-pocket cost burden, out-of-pocket costs, cancer, survey data, cancer registry data, equity

## Abstract

Background: The burden of out-of-pocket costs among cancer patients/survivors in Canada is not well understood. The objective of this study was to examine the health-related out-of-pocket cost burden experienced by households with a cancer patient/survivor compared to those without, examine the components of health-related costs and determine who experiences a greater burden. Data and methods: This study used a data linkage between the Survey of Household Spending and the Canadian Cancer Registry to identify households with a cancer patient/survivor (cases) and those without (controls). The out-of-pocket burden (out-of-pocket costs measured relative to household income) and mean costs were described and regression analyses examined the characteristics associated with the household out-of-pocket burden and annual out-of-pocket costs. Results: The health-related out-of-pocket cost burden and annual costs measured in households with a cancer patient/survivor were 3.08% (95% CI: 2.55–3.62%) and CAD 1600 (95% CI: 1456–1759), respectively, compared to a burden of 2.84% (95% CI: 2.31–3.38) and annual costs of CAD 1511 (95% CI: 1377–1659) measured in control households, respectively. Households with a colorectal cancer patient/survivor had a significantly higher out-of-pocket burden compared to controls (mean difference: 1.0%, 95% CI: 0.18, 0.46). Among both cases and controls, the lowest income quintile households experienced the highest health-related out-of-pocket cost burden. Interpretation: Within a universal health care system, it is still relevant to monitor health-related out-of-pocket spending that is not covered by existing insurance mechanisms; however, this is not routinely assessed in Canada. We demonstrate the feasibility of measuring such costs in households with a cancer patient/survivor using routinely collected data. While the burden and annual health-related out-of-pocket costs of households with a cancer patient/survivor were not significantly higher than control households in this study, the routine measurement of out-of-pocket costs in Canada could be systemized, providing a novel, system-level, equity-informed performance indicator, which is relevant for monitoring inequities in the burden of out-of-pocket costs.

## 1. Introduction

Cancer will impact over 22 million people worldwide by 2030 [1]. In Canada, approximately 225,000 individuals were diagnosed with cancer in 2020 [2]. The expected rise in cancer cases due to the aging of the population and increased survivorship, coupled with new and expensive treatments, has led to the growth of cancer care expenditures and an increasing economic burden on both the health care system and individuals. Although extensive work has been done to estimate the magnitude of the cancer burden on third-party public payers, particularly public health care systems [3,4,5], there has been less focus on understanding and describing the economic burden of cancer costs for patients and their families, especially in Canada.

The existing research has focused on what is known as financial toxicity [6], defined broadly as the harmful impacts of financial strain that are linked to a cancer diagnosis, and its impact on the wellbeing of patients, families and society [7,8]. One contributing factor to financial toxicity is cancer-related out-of-pocket costs, defined as medical or non-medical direct costs paid by patients and their families that are not reimbursed. Studies from mostly high-income countries have found that out-of-pocket costs can be substantial, with an average of USD 300 per month in the US, and between USD 70 and USD 200 per month reported in studies conducted in Australia, western Europe and Canada [9]. Medications have been documented to be the highest cost category in the US [9]. In countries with national health insurance schemes that provide universal coverage of cancer care, such as Canada and Australia, non-medical costs were among the highest out-of-pocket costs faced by cancer patients [9].

In Canada, cancer patients experience many financial challenges after diagnosis [10]. A national survey conducted in 10 Canadian provinces found that 44% of cancer patients reported financial problems, such as being unable to pay health care bills [11]. In another study, 33% of patients reported high levels of financial burden, and patients who reported the “worst burden” spent approximately 50% of their monthly income on out-of-pocket costs [12]. Most of the existing Canadian out-of-pocket cost studies have focused on patients in selected regions, or with specific cancers [13,14,15,16], or on regional clinic-based programs with limited generalizability beyond the study context [17,18,19].

The objectives of this study were to examine the health-related out-of-pocket cost burden experienced by households with a cancer patient/survivor compared to those without, examine the components of health-related costs and determine who experienced a greater burden.

## 2. Data and Methods

### 2.1. Study Design and Setting

This study made use of a retrospective, matched case-control design to examine the health-related out-of-pocket cost burden and annual costs of households with a cancer patient/survivor (cases) and those households without (controls), and to identify the characteristics associated with that burden. To create the study cohorts, the 2010–2017 waves of the Survey of Household Spending (SHS) and the 2005–2017 Canadian Cancer Registry (CCR) were linked using basic personal identifiers within the Social Data Linkage Environment [20] and the Derived Record Depository (DRD) at Statistics Canada. Records from the SHS and CCR, which link the same individual within the DRD, are considered to be successfully linked. Detailed information about the SDLE and the DRD can be found elsewhere [20].

### 2.2. Data

The SHS is designed to be a nationally representative population survey administered to a target population, which includes Canada’s 10 provinces and the territorial capitals. The sampling excludes residents of institutions, all individuals residing on military camps, and people living on reserves (i.e., about 2% of the total population). It is administered annually to approximately 48,570 households across Canada [21] and collects detailed information on household expenditures, annual self-reported household income, the demographic characteristics of the household, dwelling characteristics (e.g., type, age and tenure) and household equipment owned (e.g., electronics and communications equipment). The SHS includes a questionnaire and an expenditure diary. The questionnaire is used to collect regular and less frequent expenses using a computer-assisted personal interview. The expenditure diary is only provided to a subsample of SHS household respondents and is used to collect frequent or smaller expenses, which can be difficult to recall during a retrospective interview. For the present study, only information collected via the questionnaire was examined. Our objective was to examine and compare the health-related out-of-pocket burden and costs in a sample of households with and without a cancer patient/survivor. As our goal was not necessarily to produce nationally representative results, survey weights were not used in the SHS in this study. There is a potential for non-response bias in the unweighted SHS and, thus, the expenditure data are representative of this sample and not nationally representative.

The CCR is a national population-based cancer registry, which includes data collected and reported to Statistics Canada by each provincial/territorial cancer registry on new primary cancers diagnosed among Canadian residents since 1992 [22]. From 2011 onwards, Quebec has not reported data to the CCR. The CCR and, thus, this cohort only includes cancer cases from Quebec up to 2011.

**Figure 1:**
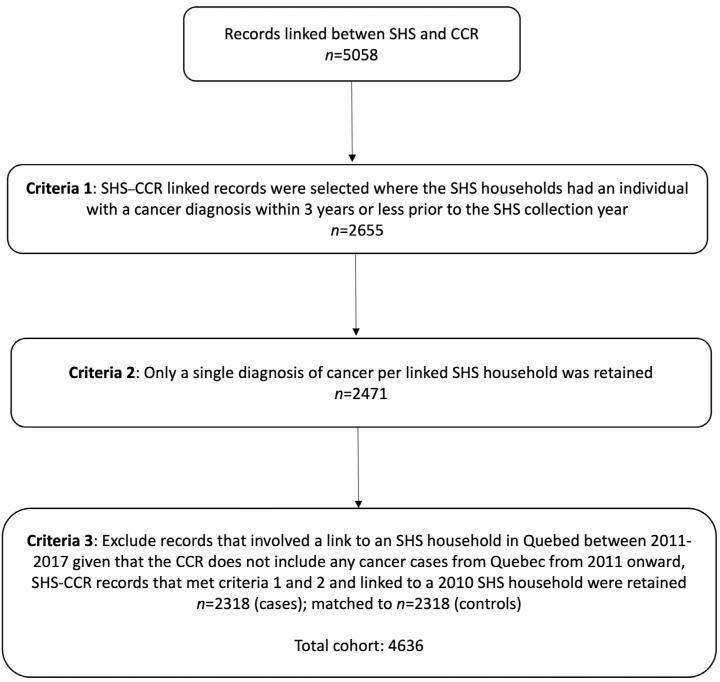
Study cohort inclusion criteria.

The SHS and CCR were linked to the DRD using standard deterministic (i.e., exact matching) and probabilistic record linkage methodologies. For this linkage, all members of responding households from the 2010–2017 SHS annual waves were combined to create a cohort of 168,631 unique individuals who were eligible for linkage to the DRD. The variables used in the linkage included birthdate, sex, given names, surnames, postal code of residence, city, Census Metropolitan Area, Census Subdivision, province and telephone number. Overall, 96.6% (162,928/168,631) of the eligible SHS household members were successfully linked to the DRD. In the 1992–2017 CCR files, 3,665,898 records were eligible for linkage to the DRD, with a 99.8% linkage rate. The outcome file for this analysis contained 5082 successful linkages at the household level between the SHS and CCR records, referred to hereafter as SHS-CCR records (Figure 1). We note that this linked cohort is not nationally representative, and excludes key populations (Quebec residents from 2011 onwards, residents of military camps and First Nations peoples living on reserves).

### 2.3. Population

Cases were defined as all households that included an individual diagnosed with cancer within a maximum of three years prior to the SHS collection year. SHS households selected for this linkage had at least one cancer diagnosis within 3 years of SHS collection. This time frame was selected to allow for a sufficient sample size for the analysis. Where there was >1 cancer diagnosis in the household, the person with the cancer diagnosis closest to the date of SHS collection was retained in the linkage. The final case sample included 2318 records (i.e., case households).

Controls were defined as 2010–2017 SHS households that did not include an individual diagnosed with cancer (i.e., not linked to the CCR). The controls were hard matched 1:1 to cases on sex (female, male), age (±2 years), SHS survey year, household composition (e.g., number of adults and children) and the first three digits of the provincial or territorial postal code. Where multiple SHS control households were identified, one household was chosen at random to act as a control.

### 2.4. Outcomes

The primary outcome of the analysis was the health-related out-of-pocket cost burden, defined as the ratio of annual health-related out-of-pocket costs to annual household income (before taxes, adjusted by household size (the household size adjustment was calculated as the square root of n, number of household members), expressed as a percentage.

The secondary outcome was the annual health-related out-of-pocket costs incurred by the households. The health-related out-of-pocket costs were based on items collected by the SHS questionnaire, which did not change between 2010 and 2017 and were calculated as the sum of health care services, medicines, and eye and dental care (Table 1). Transportation-related costs were not included as there was no way to discern whether these were for health-related or other reasons, such as leisure. All costs were reported in 2019 constant Canadian dollars using the appropriate Consumer Price Index for Health and Personal Care [23].

Both outcomes were measured using annual costs over the same period. The two outcomes are distinct, yet complementary. While annual health-related costs allow us to understand how much households are spending and in which categories, the health-related out-of-pocket cost burden allows us to assess the affordability of this expenditure relative to the household’s resources (i.e., income).

### 2.5. Variables

The age and sex information for the cases came from the CCR, while the age and sex information for the controls came from the SHS. All household-level variables for the cases and controls came from the SHS and included the following variables: household size (total number of persons in the household), household type (number of household members and the relationship of household members, e.g., single, married, children), income expressed in quintiles (derived at the national level, from household income before taxes in the year prior to the SHS reference year, adjusted for household size), home ownership (yes/no), private health insurance (yes/no), urban size of place of residence (rural area; population centre 1000 to 99,999; population centre 100,000 to 999,999; population centre 1,000,000 or over) and regional groupings (1: British Columbia and Alberta; 2: Saskatchewan and Manitoba; 3: Ontario and Quebec; 4: Newfoundland and Labrador, Prince Edward Island, Nova Scotia and New Brunswick; and 5: Whitehorse, Yellowknife and Iqaluit).

Cancer type for all cases was derived from the CCR and defined according to the International Classification of Diseases (ICD)-Oncology (O) codes.

### 2.6. Descriptive Analysis

First, we summarized the case and control cohorts using means and standard deviations (SD) and proportions to describe the demographic and socioeconomic characteristics of the sample. Second, we summarized the primary and secondary outcomes for the case and control households and completed pairwise comparisons using *t*-tests, and *p*-values to examine the differences, including by demographic and socioeconomic characteristics. For the cases, the primary and secondary outcomes were also examined by the cancer diagnosis of the patient/survivor. Third, we descriptively assessed the within-group trends (i.e., trends in the outcomes within cases and control households).

### 2.7. Regression Analysis

Multivariate linear regression was used to estimate the relationship between cancer status in the household and health-related out-of-pocket cost burden (primary outcome) and annual health-related out-of-pocket costs (secondary outcome). The primary outcome was modeled using an OLS regression model that specified a normal distribution and identity link. In this model, the independent variable was modeled as a binary variable that defined cases as all households with a cancer patient/survivor and controls as households without a cancer patient/survivor. An alternate independent variable was also defined using a categorical variable that identified the household as having someone with either breast cancer, prostate cancer, lung cancer, colorectal cancer, all other cancers or no cancer (controls). The secondary outcome was modeled using a generalized linear model (GLM). Previous research has shown that GLMs can handle data with a relatively small proportion of zeros in the overall sample, as found in this study (i.e., less than 10% of households reported annual health-related out-of-pocket costs = CAD 0) and are the preferred model for estimating expenditure outcomes [24,25]. The GLM specified a gamma distribution and a log link. The outcome was estimated using a binary independent variable to identify case and control status.

Model estimates, adjusted for sex, regional grouping (using the group of Newfoundland and Labrador, Prince Edward Island, Nova Scotia and New Brunswick as a reference group, given it was the largest group in the sample), urban size of residence, home ownership and private health insurance, were estimated. The adjusted model for health-related out-of-pocket costs also included the household income quintile. The beta coefficients and their standard errors as well as adjusted means and 95% confidence intervals were estimated in all models.

### 2.8. Sensitivity Analyses

Given the lack of consensus in the literature on the measurement of the out-of-pocket cost burden and the expenditure items to include in the calculation of the total costs, we conducted a number of additional analyses using alternate definitions. We describe these here as sensitivity analyses that were undertaken to support the main analysis. First, the health-related out-of-pocket cost burden and annual health-related out-of-pocket costs were calculated with and without insurance premiums included, as insurance premiums are not a standard out-of-pocket cost category. This comparison allowed us to explore the impact of paying for premiums among those who reported these costs.

We also examined an alternate definition of the primary outcome, calculated as health-related out-of-pocket costs as a proportion of total expenditure, and similar results were measured using both definitions for this outcome. Given this, we used the most common definition (out-of-pocket costs as a proportion of total expenditure) as the primary outcome in this study.

All data and regression analyses were performed using SAS-callable SUDAAN (Raleigh, NC, USA), version 11.0.3. All estimates are unweighted.

## 3. Results

### 3.1. Population Characteristics

There were 4636 case-control households included in the study. The median age of the respondents was 65.5 years and there was a roughly similar representation of households included from the following regional groupings: British Columbia and Alberta (22.8%), Saskatchewan and Manitoba (19.1%) and Ontario and Quebec (19.8%). The remaining households were located within the regional groups that included Newfoundland and Labrador, Prince Edward Island, Nova Scotia and New Brunswick (the over-representation of households from the Atlantic provinces may result from a higher response to the SHS in this region of Canada) (38.7%) and Whitehorse, Yellowknife and Iqaluit (0.7%) (Table 2). Over three-quarters of households (78.4%) were located in urban centers and over half of the households included two individuals (57.9%) residing as a couple. A total of 39.6% of households had an annual income less than CAD 50,000 and 26.1% of households reported an annual income greater than CAD 100,000. Home ownership was high (81.7%), and under half of households reported having supplementary private health insurance (45.4%).

The cases were distributed evenly across diagnosis years, with 32.9% of the cancer households having an individual who was diagnosed two to three years prior to the SHS survey year, 33.8% within 1–2 years and 33.3% within 0 to 1 year of diagnosis (Table 2). The most common cancer among the cases was breast cancer (18.0%), followed by colorectal cancer (14.0%) and prostate cancer (13.8%). We grouped all cancers that were individually present in less than 1% of the sample; jointly, households with a patient/survivor with these cancers represent 26.2% of the total sample.

### 3.2. Health-Related Out-of-Pocket Burden and Annual Health-Related Out-of-Pocket Costs among Case Households

The highest burden was measured in households with a colorectal cancer patient/survivor (3.7%, SD: 8.6), followed by households with individuals with the following cancer diagnoses: lung and bronchus (3.4%, SD: 4.5), bladder (3.3%, SD: 4.0), uterine (3.0%, SD: 3.0, SD: 7.0), non-Hodgkin’s lymphoma (2.3%, SD: 3.4), prostate (2.5%, SD: 3.4%) and melanoma (2.3%, SD: 3.5) (Table 3). In contrast, the lowest health-related annual out-of-pocket costs were measured in households with a colorectal cancer patient/survivor (CAD 1530; SD: CAD 2072) and the highest costs were reported among those with a melanoma diagnosis (CAD 1898, SD: CAD 3487).

### 3.3. Health-Related Out-of-Pocket Burden and Costs in Households with and without a Cancer Patient/Survivor

In Table 3, we show the pairwise comparison of the health-related out-of-pocket cost burden and annual health-related out-of-pocket costs by the demographic and socioeconomic characteristics of the case and control households. When the outcomes were examined by income quintile, regional group, size of urban place of residence, home ownership and private health insurance status, we measured marginally higher health-related out-of-pocket cost burdens and annual health-related costs for households with a cancer patient/survivor, but none of the differences reached statistical significance (at the level of *p* < 0.05). The one exception was that cases in the regional group that included Newfoundland and Labrador, Prince Edward Island, Nova Scotia and New Brunswick reported higher annual health-related out-of-pocket costs compared to the controls (CAD 1454 (SD: CAD 1885) vs. CAD 1277 (SD: CAD 1780); *p* = 0.04).

Among both cases and controls, the lowest income quintile households experienced the highest health-related out-of-pocket burden (cases: lowest: 5.9% (SD: 12%) vs. highest: 1.2% (SD: 2.2%); controls: lowest: 5.3% (11.7) vs. highest: 1.1% (SD: 1.6%)). In contrast to the out-of-pocket burden, both the case and control households in the highest income quintiles had greater out-of-pocket costs compared to those in the poorest income quintiles (cases: lowest: CAD 1187 (SD: 2448) vs. highest: CAD 2240 (SD: 3978); controls: lowest: CAD 1083 (SD: 1858) vs. highest: CAD 1963 (SD: 2831). For the cases and controls, both the health-related out-of-pocket cost burden and annual health-related out-of-pocket costs were greatest for households in the regional group that included Saskatchewan and Manitoba (3.8% (SD: 7.9); CAD 2173 (SD: CAD 3459), respectively) and lowest in the regional grouping that included Whitehorse, Yellowknife and Iqaluit (3.8%, SD: 7.9; CAD 868 (SD: CAD 1418, respectively). Both home ownership and private health insurance, proxies for wealth, showed a consistent pattern for the cases and controls: a lower burden in households reporting home ownership (and private health insurance), but higher annual costs.

In the unadjusted analyses, there was a non-significant difference in the health-related out-of-pocket burden and mean health-related out-of-pocket costs between the cases and controls (health-related burden: mean difference: 0.1; 95% CI: −0.2, 0.5, *p*-value: 0.4; annual health-related out-of-pocket cost: mean difference CAD 88; 95% CI: −56,233, *p*-value = 0.2) (Table 4). The cases reported higher spending on most health-related cost categories compared to the controls, but only the expenditure on medicines was significantly higher for the cases (mean difference: CAD 85; 95% CI: 1168, *p* = 0.05).

### 3.4. Regression Analysis

The model estimated a non-significant relationship between having a cancer patient/survivor in the household and the household’s out-of-pocket burden, accounting for all other covariates (β: 0.24, SE: 0.19, *p* = 0.2) (Table 5). When the independent variable was disaggregated by cancer-type, the model estimated a positive relationship between households with a colorectal cancer patient/survivor and the health-related out-of-pocket burden outcome that approached significance. This entailed a marginal difference in the adjusted means of 0.8 percentage points for colorectal cancer cases compared to the controls (3.62% vs. 2.84%). All covariates, with the exception of the variable for sex, had a significant association with the out-of-pocket burden outcome (Table 6).

For the secondary outcome, the model estimated a non-significant relationship between cases and health-related out-of-pocket costs (β: 0.08; SE: 0.06, 0.7) (Table 7).

### 3.5. Sensitivity Analyses

The inclusion of health insurance premiums in the calculation of the primary and secondary outcomes did not substantively alter the main findings.

## 4. Discussion

This study involved the first-ever linkage of the Canadian Cancer Registry with the Survey of Household Expenditure to examine the burden of health-related spending for households with a cancer patient/survivor compared to households not impacted by cancer. While any cancer diagnosis in the household was not significantly associated with a household’s health-related out-of-pocket burden, we found a positive, yet non-significant association for households with a colorectal cancer patient/survivor. Likewise, there was no association found between the cases and annual out-of-pocket costs. This study offers an important contribution to a small, but growing body of literature on health-related out-of-pocket costs in Canada by supplementing an evidence base that has predominantly examined out-of-pocket costs using cross-sectional patient surveys.

While none of the previous studies on this topic in Canada have employed a case-control design, the level and magnitude of out-of-pocket costs measured in the current study are consistent with these previously reported estimates. A systematic review found that monthly out-of-pocket costs for cancer in Canada ranged from USD 15 to 400 per month [9]. A recent study of patients actively receiving cancer treatments reported a mean 28-day out-of-pocket cost of CAD 518; in the study, patients spent about 15.1% of their monthly income on out-of-pocket expenses [8]. At the lower end, Lauzier et al. (2013) measured annual medical and non-medical out-of-pocket costs in a national study of breast cancer patients and found these costs accounted for 2.3% of annual household income [14]. The variability in estimates between studies reflects differences in the populations studied and the approaches used to measure and report on out-of-pocket costs. For example, most patients in our study were diagnosed with cancer more than 1 year prior to the completion of the SHS, whereas the patients in Longo et al. (2020) included patients actively receiving treatments [8]. Another US study that reported out-of-pocket costs across care phases (initial, continuing, end-of-life) showed that out-of-pocket costs were highest during the initial and end-of-life phases (USD 2443 and USD 4271, respectively) and lowest in the continuing phase (USD 593), where many patients were not actively receiving treatments. In addition, methodological differences in cost categories, recall periods, approaches to measurement and the estimation of the cost burden also pose challenges for comparing findings across studies [26,27]. While our study demonstrates that households impacted by cancer do not necessarily face higher costs than control households, the levels of spending can still impact significantly on households, which is an area that merits greater policy attention.

This work is timely and relevant, as it also serves to demonstrate the feasibility of leveraging an existing dataset to support more routine monitoring of an indicator that is relevant for understanding equity in cancer systems and health systems. Through this linkage, it may be possible to examine and track the out-of-pocket cost burden of cancer populations by income group or geographical region, as well as other relevant individual and household characteristics. This would provide a much-needed system performance indicator to better understand and describe the profile of out-of-pocket costs and their burden in Canada, including disparities, drivers of costs and the impact of policy and program interventions on patients’ health-related costs over time. Building on this linkage would support a more equity-informed approach to system performance monitoring, as called for in the Canadian Strategy for Cancer Control [28].

### Strengths and Limitations

The strengths of this study include the use of a case-control design and a sample of Canadian households drawn from a national survey. The cases and controls were hard-matched according to several variables, which enabled a comparison of the outcomes in similar households, which has not been examined in the Canadian cancer literature.

Nonetheless, this study has limitations. The control households were selected from the SHS, which does not include questions on the health status of the household respondents. By matching according to age, we attempted to account for the association between age and co-morbidity across the cases and controls, particularly as cancer is known to be associated with other comorbidities. While the control households did not include a household member with a cancer diagnosis within the observed timeframe, it was not possible to rule out other acute or chronic conditions that could have impacted their health-related out-of-pocket costs. This points to an area for further development of the SHS, such as the inclusion of a health module that would further enrich the understanding of the household context. Due to the limited sample size, we did not include data from the SHS diaries, which included detailed information on expenditures, including more nuanced information on transportation and travel, which can be a key source of economic burden for cancer patients, especially those who are rural-residing. In addition, two thirds of the study population were diagnosed with cancer greater than 1 year prior to completing the SHS. Out-of-pocket costs are much higher during the initial (e.g., while undergoing active treatment) and terminal care phases [29], which may be under-represented in this study population. Thus, this work offers a conservative estimate of the out-of-pocket cancer burden in Canada. We only examined the 2010–2017 SHS waves (i.e., the most recent waves at the time of the study) and did not include survey weights. This limited our sample size and, thus, our ability to examine the data by individual provinces and territories. We addressed this by grouping the provinces and territories by region, which potentially masks context-specific differences. Further, the data were not nationally representative (and excluded residents of institutions, all individuals residing on military camps and people living on reserves—about 2% of the population) and, thus, reflect the burden of health-related out-of-pocket costs and annual costs for the study cohort. Moreover, Quebec was under-represented in the linked cohort as the CCR does not include data from Quebec from 2011 onwards. However, there are plans underway to update the CCR with data from Quebec. Through this linkage, future research could expand the size and representativeness of the cohort to explore, using a nationally representative sample, how the burden is distributed across the population, including by province and territory and by major socioeconomic groupings.

## 5. Conclusions

In this case-control study, we found no differences between the burden of health-related out-of-pocket costs and annual costs between households with a cancer patient/survivor and control households. This research suggests that while cancer households may not be worse off in terms of the burden of out-of-pocket costs that are experienced, attention to monitoring out-of-pocket costs in Canada can provide important insights on how this burden may change over time and the cancer populations who might be most affected. We have demonstrated the feasibility of using routinely collected data to measure health-related out-of-pocket cost and, thus, this work sets the stage for systematizing the ongoing monitoring of health-related out-of-pocket costs and their burden in cancer populations over time, with the potential to expand to other disease populations. This linkage will be key for tracking health system progress on ensuring equity in health financing for cancer in Canada.

## Figures and Tables

**Table 1:** Items included in expenditure categories.

Expenditure Categories	Reference Periods
Care/services expenditures are the sum of:	
Health care practitioners in the home	12 months
Other health care practitioners	12 months
Health care by general practitioners and specialists	12 months
Weight control programs, smoking cessation programs and other medical services	12 months
Hospital care, nursing homes and other residential care facilities	12 months
Medicines are the sum of:	
Prescribed medicines and pharmaceutical products	3 months
Eye and dental are the sum of:	
Prescription eye wear	12 months
Eye-care services (e.g., surgery, exams)	12 months
Dental services	12 months
Insurance * is the sum of:	
Private health care plan premiums	12 months
Dental plan premiums	12 months
Accident or disability insurance premiums	12 months

* Note: Insurance expenditures were not included in the analysis reported. Transportation-related expenditures are presented separately and are not included in health-related out-of-pocket burden or estimates because there was no way to discern whether the reported transportation costs were for health-related reasons.

**Table 2:** Characteristics of respondents and households with and without cancer.

	Households with Cancer (Cases)	Matched Households without Cancer (Controls)	
Characteristic	*n* (SD/%)	*n* (SD/%)	*p*-Value
**Number**	2318	2318	
**Age, mean (SD)**	65.5 (SD:12.8)	65.5 (SD:12.7)	0.864
**Age group, years**			0.964
0–39	80 (3.5%)	81 (3.5%)	
40–54	348 (15.0%)	350 (15.1%)	
55–64	574 (24.8%)	566 (24.4%)	
65–74	725 (31.3%)	746 (32.2%)	
75+	591 (25.5%)	575 (24.8%)	
**Sex**			1
Female	1228 (53.0%)	1228 (53.0%)	
Male	1090 (47.0%)	1090 (47.0%)	
**Regional groups**			1
Newfoundland and Labrador, Prince Edward Island, Nova Scotia and New Brunswick	898 (38.7%)	898 (38.7%)	
Ontario and Quebec	433 (18.7%)	433 (18.7%)	
Saskatchewan and Manitoba	442 (19.1%)	442 (19.1%)	
British Columbia and Alberta	528 (22.8)	528 (22.8%)	
Whitehorse, Yellowknife and Iqaluit	17 (0.7%)	17 (0.7%)	
**Household Type**			1
One-person household	482 (20.8%)	482 (20.8%)	
Couples without children	1225 (52.8%)	1225 (52.8%)	
Couples with children	360 (15.5%)	360 (15.5%)	
Couples with other related or unrelated persons	82 (3.5%)	82 (3.5%)	
Lone-parent household with no additional persons	74 (3.2%)	74 (3.2%)	
Other household with related or unrelated persons	95 (4.1%)	95 (4.1%)	
**Income**			0.061
1st (lowest) income quintile	442 (19.1%)	487 (21.0%)	
2nd	455 (19.6%)	474 (20.4%)	
3rd	501 (21.6%)	426 (18.4%)	
4th	467 (20.1%)	461 (19.9%)	
5th (highest) income quintile	453 (19.5%)	470 (20.3%)	
**Owns house**			0.819
Yes	1891 (81.6%)	1897 (81.8%)	
No	427 (18.4%)	421 (18.2%)	
**Private Health Insurance**			0.059
Yes	1085 (46.8%)	1021 (44.0%)	
No	1233 (53.2%)	1297 (56.0%)	
**Urban size of place of residence**			0.081
Rural areas	464 (20.0%)	535 (23.1%)	
Population centre 1000 to 99,999	615 (26.5%)	585 (25.2%)	
Population centre 100,000 to 999,999	722 (31.1%)	710 (30.6%)	
Population centre 1,000,000 or over	517 (22.3%)	488 (21.1%)	
**Total current consumption in CAD, 2019 constant** **dollars (1), mean (SD)**	45,503 (SD: 35,271)	45,212 (SD: 34,836)	0.741
**Total expenditure in CAD, 2019 constant dollars (2), mean (SD)**	68,092 (SD: 82,271)	67,040 (SD: 77,172)	0.623
**Case-specific characteristics**			
**Time between diagnosis year and survey completion**			
0 to 1	772 (33.3%)		
1 to 2	784 (33.8)		
2 to 3	762 (32.9)		
**Cancer type (3)**			
Breast	417 (18.0%)		
Colorectal	325 (14.0%)		
Prostate	320 (13.8%)		
Melanoma	161 (6.9%)		
Lung and bronchus	141 (6.1%)		
Bladder	130 (5.6%)		
Non-Hodgkin’s lymphoma	128 (5.5%)		
Uterus	89 (3.8%)		
All other cancers	607 (26.2%)		

(1) Total current consumption includes total spending collected via the SHS questionnaire on: food from stores, shelter, household operations, household furnishings and equipment, clothing and accessories, transportation, health-related, personal care, recreation, reading materials and other printed matter, education, miscellaneous. The items that comprise the categories could have varied from one SHS collection year to the next. (2) Total expenditure is the sum of all of the total current consumption collected via the SHS questionnaire (spending on food from stores, shelter, household operations, household furnishings and equipment, clothing and accessories, transportation, health-related, personal care, recreation, reading materials and other printed matter, education, miscellaneous) plus total expenditure collected via the SHS questionnaire (spending on income taxes, personal insurance premiums and retirement or pension fund contributions, gifts of money and support payments and charitable contributions). The items that comprise the categories could have varied from one SHS collection year to the next. (3) Cancer types were defined as: breast: ICD-O-2/3 topography C50, prostate: ICD-O-2/3 topography C619, lung and bronchus: ICD-O-2/3 topography C34, colorectal: ICD-O-2/3 topography C18, C19, C20, C26.0, melanoma: ICD-O-2/3 topography C44 and TICD_O3H histology 8720:8790, non-Hodgkin’s lymphoma: TICD_O3H histology 9590–9597, 9670–9719, 9724–9729, 9735, 9737, 9738 and 811–9818, 9823, 9827, 9837 excluding ICD-O-2/3 topography C420, C421 and C424, bladder: ICD-O-2/3 topography C67, uterus: ICD-O-2/3 topography C54, C55; household size adjustment was calculated as the square root of n (household size); legend: CAD—Canadian dollars, SD—standard deviation.

**Table 3:** Summary of out-of-pocket cost burden and out-of-pocket expenditures by household characteristics and cancer type (cases-only).

	Out-of-Pocket Cost Burden (% Income)			Annual Out-of-Pocket Costs		
Costs	Households with Cancer Patient/Survivor (Cases)	Matched Households without Cancer Patient/ Survivor (Controls)	*p*-Value	Households with Cancer Patient/ Survivor (Cases)	Matched Households without Cancer Patient/ Survivor (Controls)	*p*-Value
	Mean (SD)	Mean (SD)				
**Income quintile**						
1st quintile (poorest)	5.9 (12.0)	5.3 (11.7)	0.4	1187 (2448)	1083 (1858)	0.5
2nd quintile	4.0 (6.4)	3.8 (6.7)	0.6	1679 (2367)	1671 (2872)	0.9
3rd quintile	2.6 (3.5)	2.3 (3.4)	0.2	1694 (2364)	1490 (2009)	0.2
4th quintile	1.7 (2.4)	1.9 (2.4)	0.2	1656 (2268)	1830 (2311)	0.2
5th quintile (richest)	1.2 (2.2)	1.1 (1.6)	0.4	2240 (3978)	1963 (2831)	0.2
**Region**						
Newfoundland and Labrador, Prince Edward Island, Nova Scotia and New Brunswick	2.9 (4.5)	2.6 (6.1)	0.2	1454 (1885)	1277 (1780)	0.04
Ontario and Quebec	2.9 (8.7)	2.3 (5.9)	0.2	1533 (2659)	1359 (2085)	0.3
Saskatchewan and Manitoba	3.8 (7.9)	3.0 (4.2)	0.06	2173 (3459)	1835 (2430)	0.09
British Columbia and Alberta	2.9 (6.1)	3.8 (9.2)	0.06	1858 (3385)	2168 (3381)	0.1
Whitehorse, Yellowknife and Iqaluit	1.6 (4.0)	1.3 (2.2)	0.8	868 (1418)	1696 (2124)	0.2
**Urban size of place of residence**						
Rural areas	3.4 (5.0)	3.6 (8.4)	0.6	1556 (1842)	1515 (1864)	0.7
Population centre 1000 to 99,999	3.3 (6.4)	2.9 (5.7)	0.3	1664 (2718)	1538 (2626)	0.4
Population centre 100,000 to 999,999	2.8 (5.8)	2.4 (5.2)	0.2	1702 (2724)	1636 (2656)	0.6
Population centre 1,000,000 or over	3.0 (8.6)	2.8 (7.3)	0.7	1840 (3495)	1739 (2414)	0.6
**Owns house**						
Yes	2.9 (6.0)	2.7 (5.8)	0.3	1816 (2949)	1717 (2532)	0.3
No	3.6 (8.6)	3.8 (9.5)	0.7	1149 (1669)	1099 (1855)	0.7
**Private health insurance**						
Yes	2.5 (5.0)	2.5 (5.7)	1	1814 (3095)	1692 (2372)	0.3
No	3.6 (7.6)	3.3 (7.3)	0.3	1587 (2446)	1536 (2482)	0.6
**Cancer type**						
Colorectal	3.7 (8.6)			1530 (2072)		
Lung and bronchus	3.4 (4.5)			1752 (2342)		
Bladder	3.3 (4.0)			1758 (2208)		
Uterus	3.0 (7.0)			1784 (2436)		
Breast	2.9 (6.2)			1729 (2691)		
Non-Hodgkin lymphoma	2.6 (3.4)			1716 (2880)		
Prostate	2.5 (3.4)			1565 (1855)		
Melanoma	2.3 (3.5)			1898 (3487)		
All other cancers	3.3 (8.4)			1724 (3479)		

Cancer types were defined as: breast: ICD-O-2/3 topography C50, prostate: ICD-O-2/3 topography C619, lung and bronchus: ICD-O-2/3 topography C34, colorectal: ICD-O-2/3 topography C18, C19, C20, C26.0, melanoma: ICD-O-2/3 topography C44 and TICD_O3H histology 8720:8790, non-Hodgkin’s lymphoma: TICD_O3H histology 9590–9597, 9670–9719, 9724–9729, 9735, 9737, 9738 and 811–9818, 9823, 9827, 9837 excluding ICD-O-2/3 topography C420, C421 and C424, bladder: ICD-O-2/3 topography C67, uterus: ICD-O-2/3 topography C54, C55; income quintiles are derived within each SHS year from reported household income in current Canadian dollars adjusted for household size; household income was adjusted as the square root of n, household size; legend: SD—standard deviation.

**Table 4:** Health-related out-of-pocket cost burden, household expenditure and 12-month out-of-pocket expenditures of households with and without cancer.

	Households with Cancer	Matched Households without Cancer	Mean Difference	
Outcome	Mean (SD)	Mean (SD)	(95% Confidence Interval)	*p*-Value
**Health-related OOP cost burden ^1^, %**				
OOP costs/income	3.1 (6.9)	2.9 (6.6)	0.1 (−0.2, 0.5)	0.4
OOP costs/total expenditure	3.4 (4.7)	3.2 (4.6)	0.2 (−0.1, 0.4)	0.5
**Health-related OOP costs**				
Mean (including health insurance), CAD	2494 (3117)	2406 (2969)	88 (−79, 256)	0.3
Mean (excluding health insurance), CAD	1693 (2770)	1605 (2435)	88 (−56, 233)	0.2
**By category, CAD**				
Care and services ^2^	279 (1705)	244 (1122)	35 (−46, 116)	0.4
Medicines ^3^	832 (1517)	748 (1491)	85 (1, 168)	0.05
Eye and dental ^4^	582 (1242)	613 (1227)	−31 (−99, 37)	0.4
Insurance ^5^	800 (1264)	801 (1447)	−0.1 (−73, 72)	1

Notes: The share of expenditures was based on expenditures in constant 2019 Canadian dollars. ^1.^ Excludes insurance fees. ^2.^ Care/services are the sum of expenditures on health care practitioners in the home, other health care practitioners, health care by general practitioners and specialists, weight control programs, smoking cessation programs and other medical services, and hospital care, nursing homes and other residential care facilities. ^3.^ Medicines are expenditures on prescribed medicines and pharmaceutical products. ^4.^ Eye and dental are the sum of expenditures on prescription eyewear, eye-care services (e.g., surgery, exams), and dental services. ^5.^ Insurance is the sum of expenditures on health care plan premiums, dental plan premiums, and accident or disability insurance premiums. Legend: OOP—out-of-pcoket; SD—standard deviation.

**Table 5:** Model-adjusted out-of-pocket cost burden for households with cancer and without cancer.

	Out-of-Pocket Burden					
					95% CI	
	Beta	SE	*p*-Value	Adjusted Mean %	Low	High
**Household with cancer**	0.24	0.19	0.2	3.08	2.55	3.62
**Matched control household without cancer**	ref			2.84	2.31	3.38
**Sex**						
Male	−0.35	0.19	0.07	2.79	2.25	3.33
Female	ref			3.14	2.61	3.67
**Region**						
Ontario	−0.33	0.32	0.3	2.59	2.07	3.11
Saskatchewan and Manitoba	0.81	0.27	0.003	3.73	3.24	4.23
British Columbia and Alberta	0.73	0.31	0.02	3.66	3.18	4.14
Whitehorse, Yellowknife and Iqaluit	−1.00	1.13	0.4	1.92	−0.30	4.14
Newfoundland and Labrador, Prince Edward Island, Nova Scotia and New Brunswick	ref			2.92	2.55	3.29
**Urban size of place of residence**						
Population centre 1000 to 99,999	−0.60	0.29	0.04	2.98	−1.17	−0.04
Population centre 100,000 to 999,999	−0.95	0.28	<0.001	2.63	−1.50	−0.41
Population centre 1,000,000 or over	−0.92	0.37	0.01	2.67	−1.64	−0.19
Rural areas	ref			3.58	2.92	4.24
**Owns house**						
No	0.82	0.26	0.002	3.37	2.75	4.00
Yes	ref			2.56	2.08	3.04
**Private health insurance**						
No	0.74	0.20	<0.001	3.34	2.81	3.86
Yes	ref			2.59	2.04	3.14

Notes: Control households without cancer were derived as a hard match to cases based on: sex, age, SHS survey year, household composition and first three digits of postal code. Dollar amounts are in 2019 constant Canadian dollars. OOP health expenditure is the sum of care/services (HC006_A, HC006_B, HC007, HC008, HC009), medicines (HC003) and eye and dental (HC012, HC014, HC015) collected via the SHS questionnaire. Fully adjusted models adjust for sex, age in years, age in years squared, region, urban size of place of residence, home ownership and private health insurance. The model excludes cases and controls from Quebec. This model does not adjust for income quintiles as income is included in the outcome.

**Table 6:** Model-adjusted out-of-pocket burden for households with cancer and without cancer, by cancer type.

	Out-of-Pocket Burden					
					95% CI	
	Beta	SE	*p*-Value	Adjusted Mean %	Low	High
Household with **breast cancer**	−0.01	0.37	0.97	2.83	2.02	3.64
Household with **prostate cancer**	−0.23	0.41	0.58	2.62	1.73	3.50
Household with **lung cancer**	0.43	0.57	0.45	3.27	2.11	4.44
Household with **colorectal cancer**	0.78	0.39	0.05	3.62	2.77	4.47
Household with **“other” cancer**	0.28	0.24	0.24	3.13	2.52	3.73
**Matched control household without cancer**	ref			2.84	2.31	3.37
**Sex**						
Male	−0.34	0.21	0.11	2.88	2.30	3.46
Female	ref			3.22	2.65	3.80
**Region**						
Ontario	−0.33	0.32	0.3	2.68	2.12	3.24
Saskatchewan and Manitoba	0.81	0.27	0.003	3.82	3.28	4.35
British Columbia and Alberta	0.74	0.31	0.02	3.75	3.23	4.27
Whitehorse, Yellowknife and Iqaluit	−1.00	1.14	0.38	2.01	−0.22	4.23
Newfoundland and Labrador, Prince Edward Island, Nova Scotia and New Brunswick	ref			3.01	2.59	3.43
**Urban size of place of residence**						
Population centre 1000 to 99,999	−0.60	0.29	0.04	3.07	2.47	3.66
Population centre 100,000 to 999,999	−0.95	0.28	<0.001	2.71	2.09	3.33
Population centre 1,000,000 or over	−0.90	0.37	0.01	2.76	2.05	3.48
Rural areas	ref			3.66	2.97	4.36
**Owns house**						
No	0.81	0.26	0.002	3.46	2.80	4.11
Yes	ref			2.65	2.12	3.17
**Private health insurance**						
No	0.72	0.20	<0.001	3.41	2.86	3.97
Yes	ref			2.69	2.10	3.28

Notes: Control households without cancer patients/survivors were derived as a hard match to cases based on: sex, age, SHS survey year, household composition and first three digits of postal code. Dollar amounts are in 2019 constant Canadian dollars. OOP health expenditure is the sum of care/services (HC006_A, HC006_B, HC007, HC008, HC009), medicines (HC003) and eye and dental (HC012, HC014, HC015) collected via the SHS questionnaire. Fully adjusted models adjust for sex, age in years, age in years squared, region, urban size of place of residence, home ownership and private health insurance. The model excludes cases and controls from Quebec.

**Table 7:** Model-adjusted out-of-pocket expenditures for households with cancer and without cancer.

	Annual Out-of-Pocket Costs					
					95% CI	
	Beta	SE	*p*-Value	Adjusted Mean	Low	High
**Household with cancer**	0.08	0.06	0.17	1600	1456	1759
**Matched control household without cancer**	ref			1511	1377	1659
**Sex**						
Male	0.04	0.06	0.51	1592	1447	1751
Female	ref			1519	1385	1666
**Region**						
Ontario	−0.08	0.10	0.45	1327	1216	1449
Saskatchewan and Manitoba	0.33	0.09	<0.001	1921	1773	2081
British Columbia and Alberta	0.36	0.10	<0.001	1944	1797	2104
Whitehorse, Yellowknife and Iqaluit	−0.20	0.36	0.57	1347	906	2003
Newfoundland and Labrador, Prince Edward Island, Nova Scotia and New Brunswick	ref			1362	1280	1448
**Urban size of place of residence**						
Population centre 1000 to 99,999	0.01	0.09	0.91	1573	1427	1735
Population centre 100,000 to 999,999	0.001	0.09	0.99	1543	1393	1710
Population centre 1,000,000 or over	0.01	0.12	0.91	1583	1406	1781
Rural areas	ref			1522	1359	1704
**Owns house**						
No	−0.30	0.09	<0.001	1387	1240	1552
Yes	ref			1743	1602	1897
**Private health insurance**						
No	−0.03	0.06	0.63	1576	1436	1729
Yes	ref			1535	1394	1690
**Income quintile**						
1st quintile (lowest)	ref			1186	1062	1325
2nd quintile	0.33	0.10	<0.001	1576	1414	1756
3rd quintile	0.27	0.10	<0.001	1484	1329	1655
4th quintile	0.38	0.10	<0.001	1681	1506	1876
5th quintile (highest)	0.54	0.10	<0.001	1951	1749	2176

Notes: Control households without cancer were derived as a hard match to cases based on: sex, age, SHS survey year, household composition and first three digits of postal code. Dollar amounts are in 2019 constant Canadian dollars. OOP health expenditure is the sum of care/services (HC006_A, HC006_B, HC007, HC008, HC009), medicines (HC003) and eye and dental (HC012, HC014, HC015) collected via the SHS questionnaire. Fully adjusted models adjust for sex, age in years, age in years squared, region, urban size of place of residence, home ownership and private health insurance. Income quintiles are derived within each SHS year from reported household income in current Canadian dollars, adjusted for household size (square root of n, household size). The model excludes cases and controls from Quebec. The model dropped *n* = 317 participants from the analysis with out-of-pocket expenditures = CAD 0. When these were forced into the model using a negligible cost for each (CAD 0.0000001), the model results were consistent.

## Data Availability

The data used in this study, including the linked dataset, are available from the Statistics Canada Research Data Centres.
